# The Wiggle Index: An Open Source Bioassay to Assess Sub-Lethal Insecticide Response in *Drosophila melanogaster*

**DOI:** 10.1371/journal.pone.0145051

**Published:** 2015-12-18

**Authors:** Shane Denecke, Cameron J. Nowell, Alexandre Fournier-Level, Trent Perry, Phil Batterham

**Affiliations:** 1 School of BioSciences, Bio21 Molecular Science and Biotechnology Institute, University of Melbourne, Parkville, Victoria 3010, Australia; 2 Faculty of Pharmacy and Pharmaceutical Sciences, Monash University, Parkville, Victoria 3052, Australia; Federal University of Viçosa, BRAZIL

## Abstract

Toxicological assays measuring mortality are routinely used to describe insecticide response, but sub-lethal exposures to insecticides can select for resistance and yield additional biological information describing the ways in which an insecticide impacts the insect. Here we present the Wiggle Index (WI), a high-throughput method to quantify insecticide response by measuring the reduction in motility during sub-lethal exposures in larvae of the vinegar fly *Drosophila melanogaster*. A susceptible wild type strain was exposed to the insecticides chlorantraniliprole, imidacloprid, spinosad, and ivermectin. Each insecticide reduced larval motility, but response times and profiles differed among insecticides. Two sets of target site mutants previously identified in mortality studies on the basis of imidacloprid or spinosad resistance phenotypes were tested. In each case the resistant mutant responded significantly less than the control. The WI was also able to detect a spinosad response in the absence of the primary spinosad target site. This response was not detected in mortality assays suggesting that spinosad, like many other insecticides, may have secondary targets affecting behaviour. The ability of the WI to detect changes in insecticide metabolism was confirmed by overexpressing the imidacloprid metabolizing *Cyp6g1* gene in digestive tissues or the central nervous system. The data presented here validate the WI as an inexpensive, generic, sub-lethal assay that can complement information gained from mortality assays, extending our understanding of the genetic basis of insecticide response in *D*. *melanogaster*.

## Introduction

Insect pests are major vectors of human infectious diseases and also impose massive production losses and control costs in agriculture [1]. Chemical insecticides are often used as a weapon of choice to control or eliminate pests. While insecticides are potent in the short term, long term efficacy is often stymied by the evolution and spread of resistance [2,3]. There are a limited number of chemicals, targeting an even smaller number of insect target proteins, meaning it is critical to manage or even prevent resistance to current generation insecticides [4]. A deeper understanding of the insect’s biological response to an insecticide, and thus the array of resistance options available to insect pests, underpins rational resistance management strategies. The vinegar fly *Drosophila melanogaster*, while not a pest species, has proven to be powerful model that can be manipulated to interrogate the insect: insecticide interface [5–7].

There are many genetically determined biological mechanisms that influence how an insect responds to an insecticide. Among these, two that are particularly intensively studied because of their large contribution to insecticide resistance both in field and laboratory settings. The overexpression of genes encoding drug metabolizing enzymes (DMEs) such as cytochrome P450s (CYPs), carboxylesterases (CoEs) glutathione S transferases (GSTs) and glucuronosyltransferases (UGTs), which have the capacity to modify the chemical structure of insecticides, can cause resistance [8]. For example, the overexpression *Cyp6cm1* in *Bemisia tabaci* and *Cyp6g1* in *D*. *melanogaster* is sufficient to confer resistance to a range of insecticide classes [9–11]. The modification or loss of insecticide target sites is another commonly found and well-studied resistance mechanism. In *D*. *melanogaster* a variety of mutations in *Dα1* or *Dβ2* nicotinic acetylcholine receptor (nAChR) subunit genes confer imidacloprid resistance [12], while knockouts and amino acid substitutions in the *Dα6* subunit confer resistance to spinosad [13,14]. These findings were mirrored by studies in other insects, which found nAChR modifications linked to imidacloprid resistance in *Nilaparvata lugens* [15] and *M*. *persicae* [16] and to spinosad resistance in *Plutella xylostella* and *Frankinella occidentalis* [17,18].

Much of what is currently known about the mechanisms by which insects respond to insecticides comes from mortality assays [19], but sub-lethal insecticide response is an economically relevant phenotype that can describe additional information about insecticide biology [20,21]. Pest populations are frequently subjected to sub-lethal exposures in field settings [22,23], which have been shown to provide selection pressure [24]. For example, sub-lethal exposures to insecticides have been shown to significantly alter the fecundity [25], feeding behaviour [26] and locomotion [27] of targeted pest species. Each of these phenotypes have fitness costs and are often elicited by concentrations far below the LC_50_ value of a compound [28]. Considering these sub-lethal effects on pests may also give new insights into the mechanisms that influence insecticide resistance. Following the ingestion of an insecticide there is a cascade of biological processes which determine the response of the insect, with death as one possible endpoint. Observing the impact of sub-lethal exposures on living insects could focus on processes earlier in this cascade and the potential mechanisms of resistance that may arise from them. These points can also be extended to non-pest insects, which often undergo the same sub-lethal exposures [20]. Special attention has recently been given to the sub-lethal neonicotinoid exposures in honeybees that have been suggested to contribute to colony collapse disorder [29], although this is controversial [30].

Several methods to measure the sub-lethal effects of xenobiotics have been developed. For example, the crawling speed and pattern of *D*. *melanogaster* larvae from sensitive and control backgrounds were found to differ during acute exposure to ethanol [31]. More recently, several video tracking softwares such as Ethovision (Nodulus) and VideoTrack (ViewPoint Life Sciences) have been used to more precisely measure insect motility. These kinds of software have been used to describe sub-lethal insecticide response in mosquitos [27] and beetles [32] with a range of different insecticides. Other studies have measured alterations in sub-lethal phenotypes discussed above such as fecundity, feeding behaviour and locomotion after sub-lethal xenobiotic exposures [25–27].

Here, we describe an additional sub-lethal behavioural assay the Wiggle Index (WI; 38), which uses an open source ImageJ macro to quantify the temporal motility response of *D*. *melanogaster* larvae to sub-lethal insecticide exposures. Larvae from a wild type susceptible strain were tested with the WI with insecticides from four mode of action (MoA) classes. The response curve of the larvae differed depending on the insecticide used. The capacity of WI to detect insecticide resistance was tested using several pairs of resistant and matching control (susceptible) genotypes previously characterized using mortality assays. In each case resistant and susceptible genotypes were distinguishable. Additional biological information was also gained by observing motility response to the insecticide spinosad in a highly resistant target site mutant, suggesting additional target(s) for this compound. Using the wealth of genetic resources available in *D*. *melanogaster*, the WI can be used to investigate the role of individual genes in sublethal insecticide response.

## Methods

### Fly Stocks

All stocks were maintained at room temperature and under constant light. Armenia^14^ is an isofemale line derived from Armenia^60^ (Drosophila Genomics Resource Center #103394), and was used as a susceptible wild type for all dose response experiments. Three additional sets of genotypes consisted of lines previously associated with either imidacloprid or spinosad resistance in *D*. *melanogaster*, and had genetically matched control backgrounds (Table 1). Additionally, each of these sets had relatively well characterized resistance mechanisms that have been elaborated on in other insect species [17] or with *in vitro* studies [33,34]. Set one contains nAChR subunit mutants *Dα1* ^*M4*^ and *Dβ2* ^*L351Q*^ that were generated with EMS mutagenesis of Armenia^14^ and are resistant to imidacloprid in mortality assays [12]. *Dα1* ^*M4*^ produces a truncated *Dα1* protein product that is cut short in the transmembrane M4 domain, and *Dβ2* ^*L351Q*^ contains a single amino acid replacement in the Dβ2 subunit. Set two includes two genotypes also generated with EMS mutagenesis and selection of Armenia^14^ carrying mutations in the Dα6 nAChR subunit (*Dα6* ^*nx*^, *Dα6* ^W337*^); each confers spinosad resistance [7]. *Dα6* ^W337*^ is truncated 13 residues after the third transmembrane domain, while *Dα6* ^*nx*^ has no detectable *Dα6* expression. Set three used the HR_GAL4 to drive the expression of *Cyp6g1* in the midgut, Malpighian tubules, fat body (referred to as *digestive tissues* here) by crossing it to the UAS_Cyp6g1 line [6]. A matched control for this genotype was generated by crossing the HR_GAL4 driver line to the Φ86FB genotype, which has the same genetic background as the UAS_Cyp6g1 line [35]. Previous studies linked overexpression of *Cyp6g1* in these digestive tissues with resistance to multiple classes of insecticide [36]. Finally, in set four the overexpression of *Cyp6g1* was achieved in the central nervous system (CNS) using the Elav-GAL4 (Bloomington Stock Center # 458) driver line. Unlike the previous genotype sets, the overexpression of *Cyp6g1* in the CNS has not been previously linked to resistance.

**Table 1:** Genotypes Used in the Present Study.

Name	Background	Stock #	Resistance
*Armenia* ^*14*^	*Armenia*	*DGRC (103394)*	*Susceptible*
*Dα1* ^*M4*^	*Armenia* ^*14*^	*Batterham Lab Stock*	*Imidacloprid*
*Dβ2* ^*L351R*^	*Armenia* ^*14*^	*Batterham Lab Stock*	*Imidacloprid*
*Dα6* ^*nx*^	*Armenia* ^*14*^	*Batterham Lab Stock*	*Spinosad*
*Dα6* ^W337*^	*Armenia* ^*14*^	*Batterham Lab Stock*	*Spinosad*
*Φ86FB*	*Φ86FB*	*Bischoff et*. *al 2007*	*Susceptible*
*UAS_Cyp6g1*	*Φ86FB*	*Batterham Lab Stock*	*Imidacloprid*
*Elav-GAL4*	*Elav-GAL4*	*Bloomington (458)*	*Susceptible*

All genotypes used in the current study are displayed along with their backgrounds and previously reported resistance status.

### Obtaining Third Instar Larvae

For all isogenic lines, sixty 2–5 day old females and twenty males were collected and transferred onto maize meal medium (S1 Table) in vials sprinkled with dried yeast. For all crosses, the same procedure was followed with virgin females. In each case flies were left undisturbed for one day at 25°C for oviposition and then cleared from the vial. The vials were kept at 25°C for 68 additional hours to generate a population of early third instar larvae, which were recovered from the food using sucrose extraction [37]. Briefly, 30mL of 20% w/v sucrose (non-Analytical Reagent) in distilled H_2_0 was poured onto the food. The top layer of the food was then gently disrupted with a metal rod in order to release the larvae, which float in the sucrose solution. The solution was then carefully poured onto a fine cloth mesh to isolate the larvae, which were then dried and transferred onto grape juice agar plates (S1 Table). Third Instar larvae (~5mm in length) were then individually picked, 25 per well, into a NUNC cell culture treated 24 well plate (Thermo-Scientific) preloaded with 200μL Analytical Reagent 5% w/v sucrose (Chem Supply) in distilled H_2_0. This volume of 5% sucrose was chosen because it is the minimum amount needed to cover the 1.8cm^2^ bottom of the well. In this environment the larvae were able to translocate, but stationary gyrations represented the majority of their activity. To dose the larvae, 50μL of 5x insecticide stock solution was added to each well in order to bring the final concentration to the desired dosage. After mixing, 50μL of the mixed solution was removed in order to bring the final volume back to 200μL.

### Insecticide Dilution

Chlorantraniliprole (10 gL^-1^ Coragen®; Du Pont), Imidacloprid (200 gL^-1^ Confidor®; Bayer Crop Science), and Spinosad (10 gL^-1^ Success®; Yates) were all purchased commercially and diluted to 5,000ppm stocks using distilled water. On the day of exposure, 5x stocks were generated for each dose being used (Chlorantraniliprole: 60, 30, 15, 7.5, 3.75, 0ppm; Imidacloprid: 240, 120, 60, 30, 15, 0ppm; Spinosad: 240, 60, 30, 15, 7.5, 3.75, 0 ppm) by diluting the 5,000ppm stock in 5% Analytical Reagent sucrose (Chem Supply). A similar procedure was followed for Ivermectin (Sigma), and a 10,000ppm stock was generated using DMSO as a solvent. 5x stocks were generated (120, 60, 30, 15, 7.5, 0 ppm) by dissolving the original stock in 5% sucrose, and the highest concentration of DMSO used in dosing was added to the 0ppm solution in order to control for solvent effects, none of which were observed.

### Experimental Design

To assess the effect of different insecticides on movement, the susceptible control Armenia^14^ was tested with a range of 5 doses for imidacloprid (3ppm-48ppm), ivermectin (1.5ppm-24ppm), spinosad (1.5ppm-24ppm), and chlorantraniliprole (.75ppm-12ppm). Pilot studies were used to determine the best range of doses to be used for each insecticide. Set one mutants (*Dα1* ^*M4*^ and *Dβ2* ^*L351Q*^) were tested at 12 and 48ppm imidacloprid and the same doses were used for set two mutants (*Dα6* ^*nx*^ and *Dα6* ^W337*^) with spinosad. Set three and four genotypes expressing *Cyp6g1* in the three digestive tissues or CNS were tested at 48ppm imidacloprid.

### Filming

Ten second videos were taken at 5,10,15, 30, 45, 60, 120 and 240 minutes after the addition of insecticide and compared to videos taken immediately before the addition of the insecticide (time = 0). Each video captured larval activity in 4 individual wells that were processed separately. All filming was done using a Panasonic 3CCD Ultra-Compact™ Digital Palmcorder® against a LED light box (Huion L4S Led Light Pad). As some of the insecticides examined here are light sensitive [38,39], larvae were kept in darkness except for a brief period before and during filming. 15 seconds was allowed for larvae to acclimate to light conditions on the LED pad, before filming began. Because each 24 well plate required 6 separate videos to be taken, larvae were exposed to the light for approximately 120 seconds for each time point.

### Video Processing and Analysis of Larval Movement

**Fig 1:**
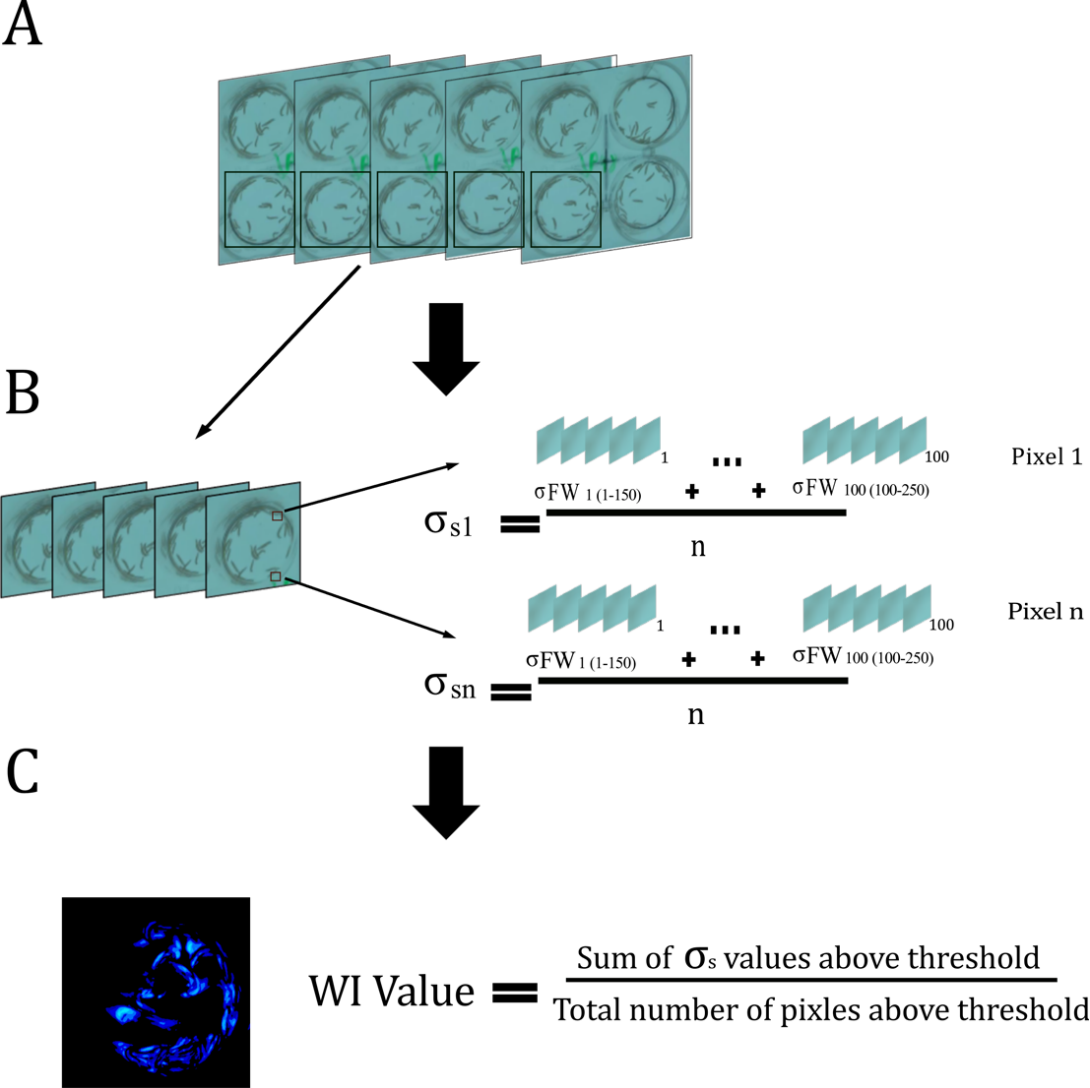
The Wiggle Index. The Wiggle Index measures total motility in a given well A) Individual wells are cropped out of a sequence of 250.jpeg images B) σ_S_ values are calculated for each pixel by calculating the standard deviation of all σ_FW_ values over the entire sequence. C) σ_S_ values from each pixel are filtered based on a threshold value and then averaged to yield the Wiggle Index (WI) value.

Raw videos were named in bulk via a renaming script written in R (R: A language and Environment for Statistical Computing). Named videos were split into jpeg image sequences using the free software Video Jpg Converter (DVDVideoSoft), and a ten second video produced 250 frames for analysis. Image Sequences were then uploaded to a server on the NeCtar research cloud for further processing. There, the Fiji distribution of ImageJ [40] was used to run the WI script (Fig 1; S1 Script).

The WI script analysed individual image sequences and calculated the total motility of the larvae in a given well. Firstly, the script crops one of the four wells from the image sequence and converts it into stacks of.tiff format (Fig 1A). An ImageJ algorithm was then performed similar to that of Preston *et*. *al* [41], which is briefly outlined here. Prior to any calculations a Gaussian Blur of 2 pixels was applied to each.tiff stack in order to minimize the impact of artefacts due to video compression. Then for each pixel, a series of frame window standard deviations (σ_FW_) was calculated by measuring the standard deviation of the pixel’s light intensity for 150 frame windows in a rolling manner starting with frame 1 (F1-F150) and continuing until the end of the stack (F101-F250). The formula is:

$${\sigma_{FW}=\left(\sqrt{\frac{1}{F}\sum_{ii=1}^{F}{\left({xf}_{i}-\mu_{W}\right)}^{2}}\right)}_{ii}$$

where W denotes the number of windows, F denotes the frame window size, μ_W_ denotes the average frame window intensity and *xf*_i_ denotes the frame intensity value. A second intermediate variable (σ_S_) was then calculated for each pixel by taking the standard deviation of the series of previously calculated series of σ_FW_ values (Fig 1B). The formula for these calculations is:

$$\sigma_{S}=\sqrt{\frac{1}{W}\sum_{i=1}^{W}{\left(-\mu_{\sigma_{W}}\right)}^{2}}$$

where μ_δW_ is the frame window intensity average.

Using the σ_S_ values for each pixel, WI values were calculated, which represent the total motility of the larvae in the ten second video. This was accomplished by averaging the σ_S_ values for every pixel above a cut-off threshold (Fig 1C). The formula for WI value is:

$$WI\;Value=\frac{\sum_{\sigma_{S}\geq T}\;\sigma_{S}}{TA}$$

where T is the threshold cut off, TA is the number of pixels above the threshold and Σ_σs≥T_ σs is the sum of values above the threshold. The cut-off threshold, in this case 30, was applied to remove any background noise due to global movement of the plate or fluctuations of the liquid.

### Visualization and Statistics

The WI script generates heat maps (Fig 1C) and reports numeric estimates of total motility (WI values) for each well at a given time. Two independent corrections were applied after the calculation of WI values to account for differences in initial motility between genotypes and to isolate the organisms’ responses to the insecticide.

**Fig 2:**
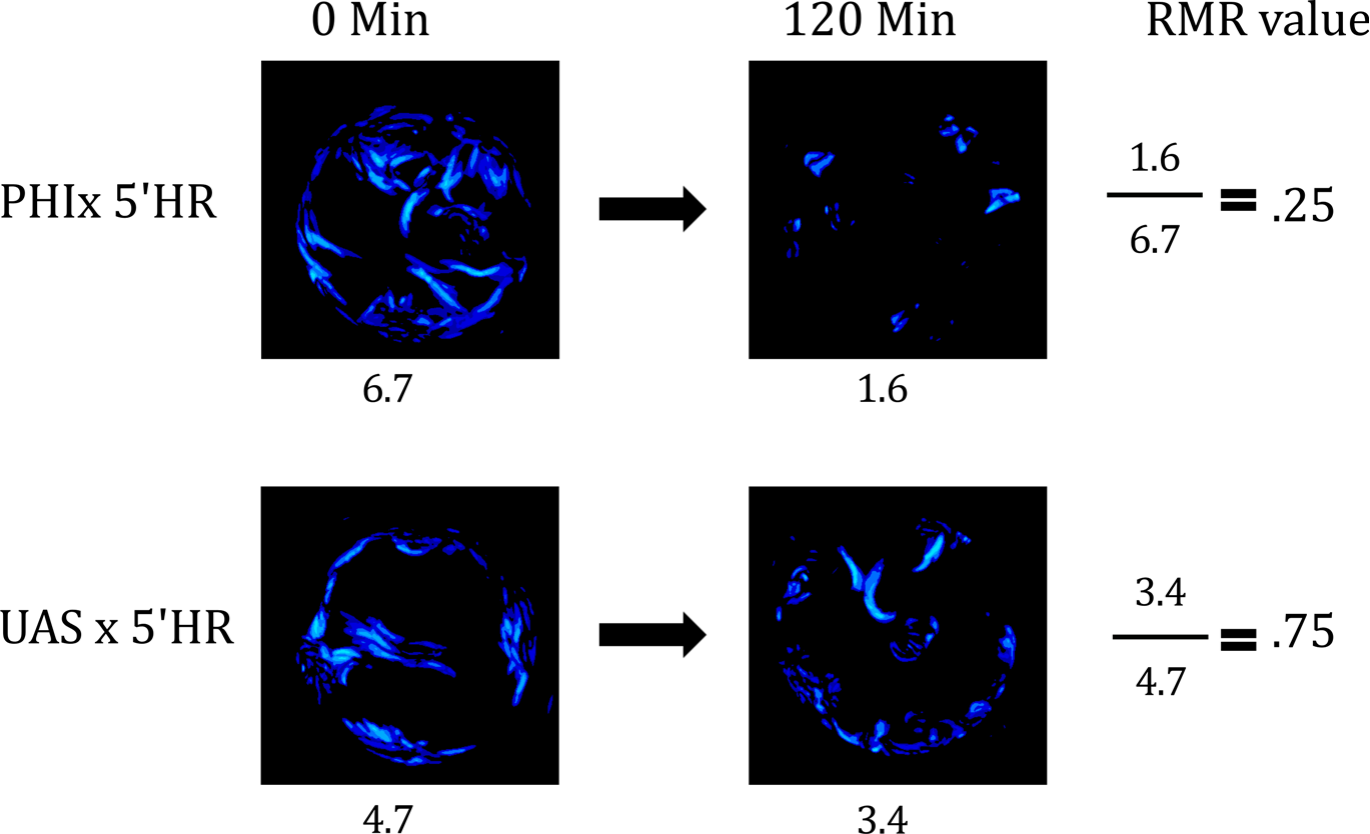
Wiggle Index Output and RMR Calculation. The Wiggle Index produced heat maps and numeric estimates of total larval motility in each well at a given time. RMR values were calculated by dividing the WI value at time = x from the WI value from the same well at time = 0.

The first correction divided WI values at a given time point by the WI value of the same larvae prior to the addition of the insecticide and generated relative movement ratio values (RMR values; Fig 2). The RMR correction normalized the motility of larvae to those same larvae in the absence of insecticide. For example, a genotype with a WI value of 10 before the addition of an insecticide and 3 at a later time point would have a RMR value of 3/10 = .3 at that time point. Mean corrected RMR values were plotted together with their associated 95% confidence intervals to visualize the response of different genotypes for a given dose or the response to different doses for a given genotype. Visualizing the data allowed for the description of several aspects of the insecticide response. The *Response Time* was defined as the time needed for a treatment to significantly and irreversibly reduce the RMR value less than one based on a 95% confidence interval. The *End Point RMR value* was defined as the final RMR value recorded (240 minutes) and was compared among doses or genotypes using Tukey’s Honestly Significant Difference (HSD) pairwise test (P≤.05).

The second correction fits a generalized linear model (GLM) to the response curve based on WI values from a particular well over time. In the case of spinosad, no transformation of the data was needed, but for imidacloprid, a log_10_ transformed time scale was used as it better fit the data based on adjusted R squared values. The inverse of the slopes of these regression lines (β values) were used as quantitative measurements of overall response. β values of each genotype were compared to control lines using a Student’s t test (P≤.05) and mean β values were plotted with 95% confidence intervals. An interactive R script *WI_Analysis* was written that accepts raw data from the WI script and generates a series of descriptive graphs including RMR curves and GLM analysis (available on GitHub https://github.com/shanedenecke/WI_Analysis).

## Results

### The Response of Wild Type to Different Insecticides

**Fig 3:**
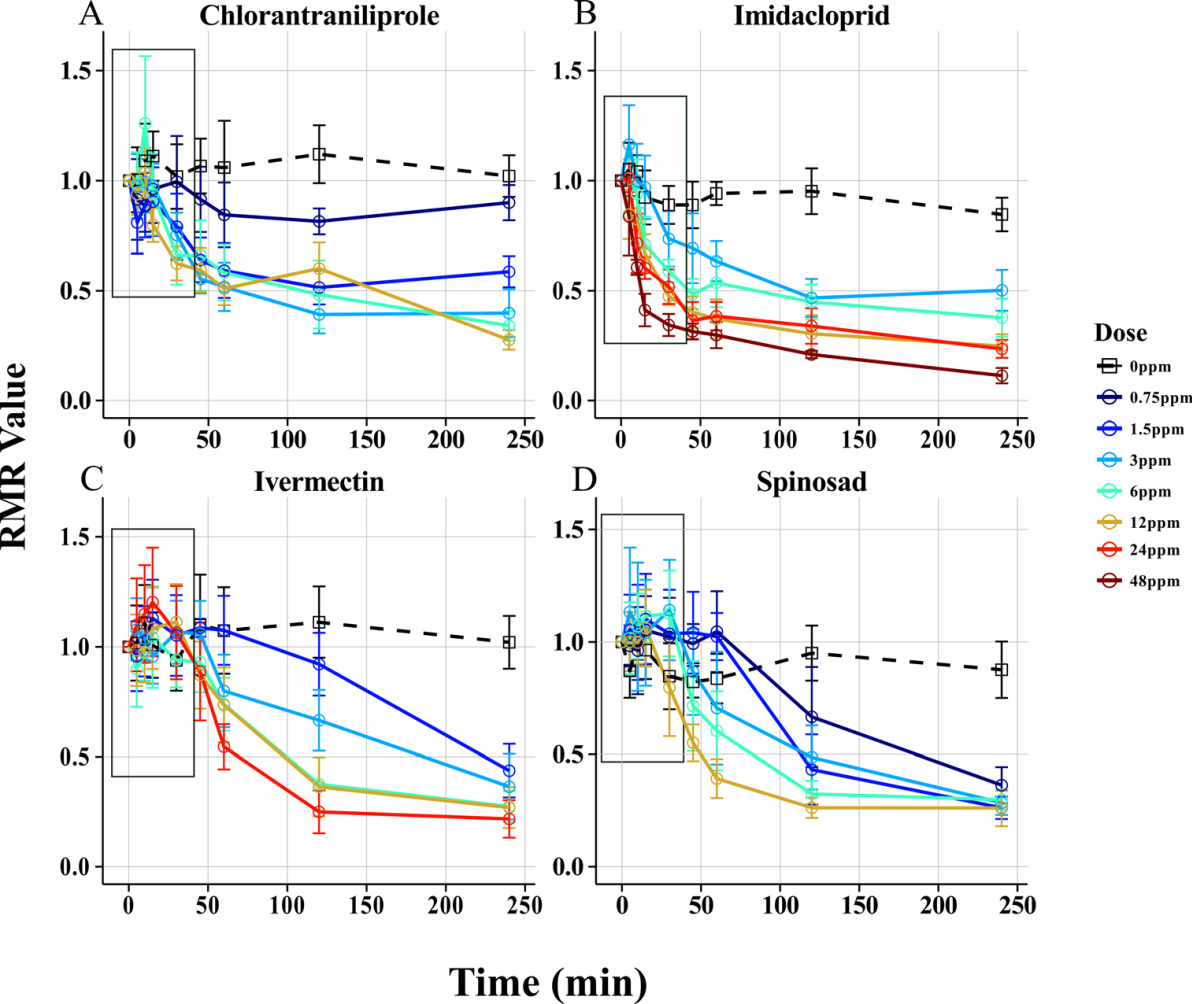
Armenia^14^ Dose Response Curves. Armenia^14^ was tested using the Wiggle Index at 5 doses of 4 insecticides. Chlorantraniliprole (A) and Imidacloprid (B) each show rapid response times and dose dependent long term RMR values. Ivermectin (C) and Spinosad (D) responded differently, displaying delayed response times and similar long term RMR values. All points correspond to the mean RMR values with 95% confidence intervals. Boxes in each plot indicate values between 0 and 30 minutes magnified in Fig 4.

**Fig 4:**
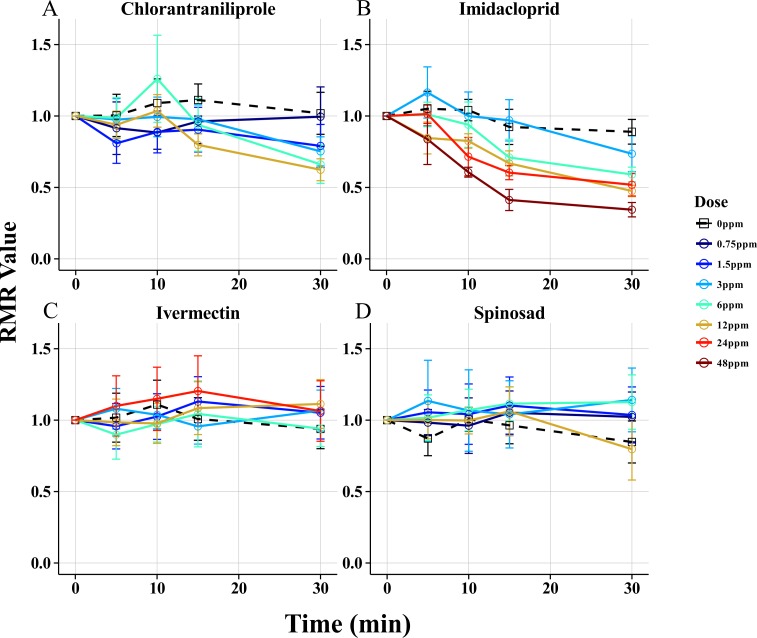
Armenia^14^ Short time Point Dose Response Curves. Armenia^14^ was tested using the Wiggle Index at 5 doses of Chlorantraniliprole (A), Imidacloprid (B), Ivermectin (C), and Spinosad (D). All points correspond to the mean RMR values with 95% confidence intervals. All measurements in this figure were observed between 0 and 30 minutes.

To observe the effect of different classes of insecticides on motility, dose response profiles were generated for Armenia^14^ with four insecticides from distinct (MoA) classes (Fig 3; Fig 4). Regardless of dose or insecticide, each chemical reduced the motility of larvae decreased as exposure time increased, but two distinct response types were observed depending on the insecticide used, *fast acting* and *slow acting*. Fast Acting responses were characterized by rapid, dose dependent Response Times and dose dependent End Point RMR values, while the Slow Acting response displayed delayed, dose dependent Response Times but with similar End Point RMR values. Imidacloprid and chlorantraniliprole were both Fast Acting, with Response Times of ten minutes or less at the highest doses tested followed by End Point RMR values that were significantly different among doses (Fig 3A and 3B; Fig 4A and 4B). Spinosad and Ivermectin were Slow Acting, with Response Times of 45 minutes or greater. The End Point RMR values for these Slow Acting insecticides were not significantly different as determined by Tukey’s HSD (Fig 3C and 3D; Fig 4C and 4D).

### Set One: Imidacloprid Resistant nAChR Alleles

**Fig 5:**
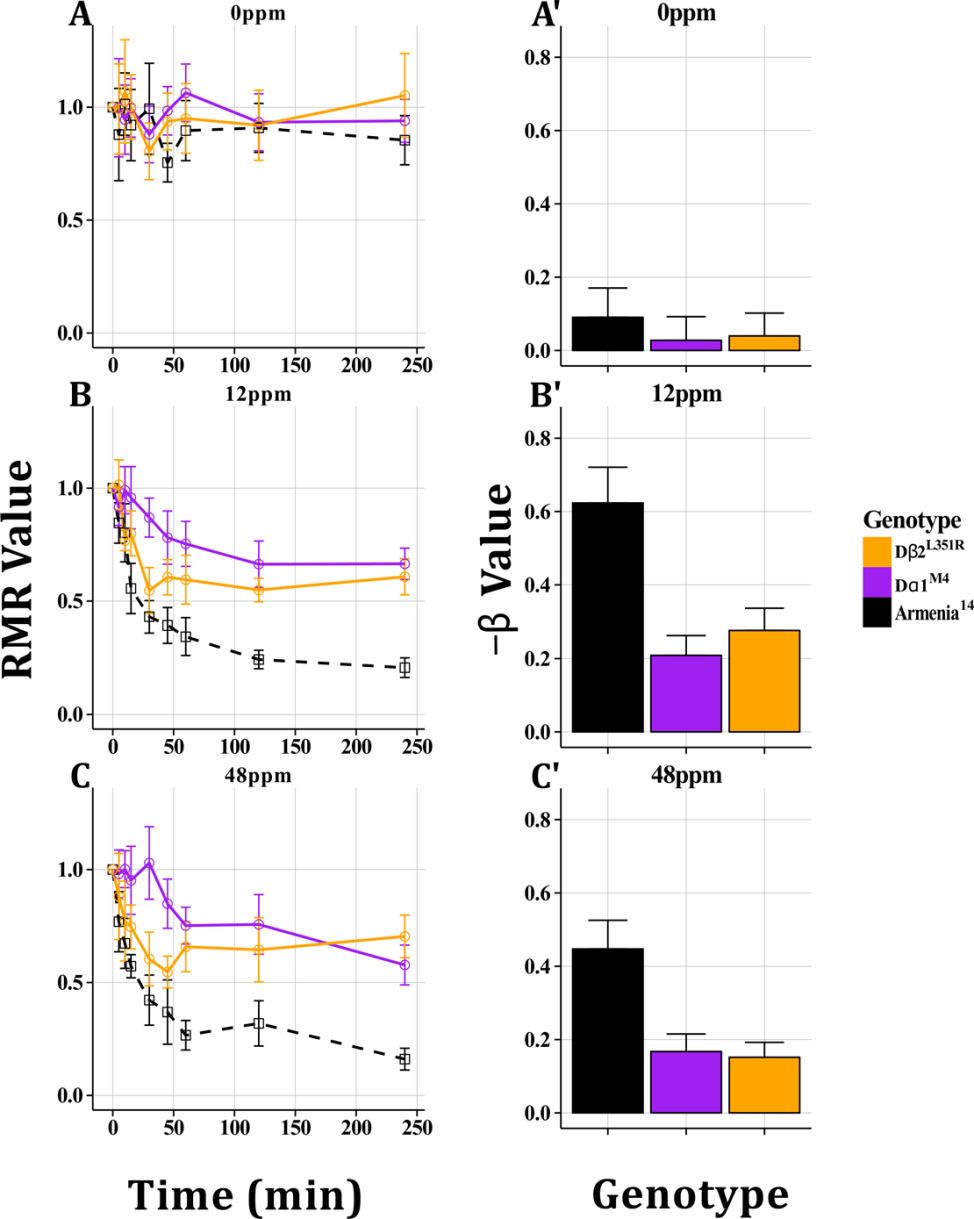
Imidacloprid Resistance in nAChR Mutants. Armenia^14^, *Dα1* ^*M4*^ and *Dβ2* ^*L351R*^ were tested using the Wiggle Index. At 0ppm (A, A’), no significant differences were observed between genotypes. As measured by Response Time, End Point RMR values and GLM analysis at 12 and 48ppm imidacloprid (B, B’, C, C’) *Dα1* ^*M4*^ responded the least, *Dβ2* ^*L351R*^ displayed an intermediate phenotype and Armenia^14^ responded the most. All plots display mean RMR or β values with 95% confidence intervals.

The motility of previously characterized imidacloprid resistant *Dα1* and *Dβ2* mutants was examined at two different concentrations of imidacloprid (12, 48ppm). Armenia^14^ showed a greater response to imidacloprid than either of the resistant mutants in the GLM analysis (Fig 5B’ and 5C’) and End Point RMR values (Fig 5B and 5C). *Dα1* ^*M4*^ had the longest Response Time and the highest End Point RMR value. *Dβ2* ^*L351Q*^ displayed an intermediate Response Time and End Point RMR Value compared to the other genotypes. In the absence of imidacloprid, there were no significant differences among genotypes in the GLM or RMR analysis (Fig 5A and 5A’).

### Set Two: Spinosad Resistant *Dα6* Alleles

**Fig 6:**
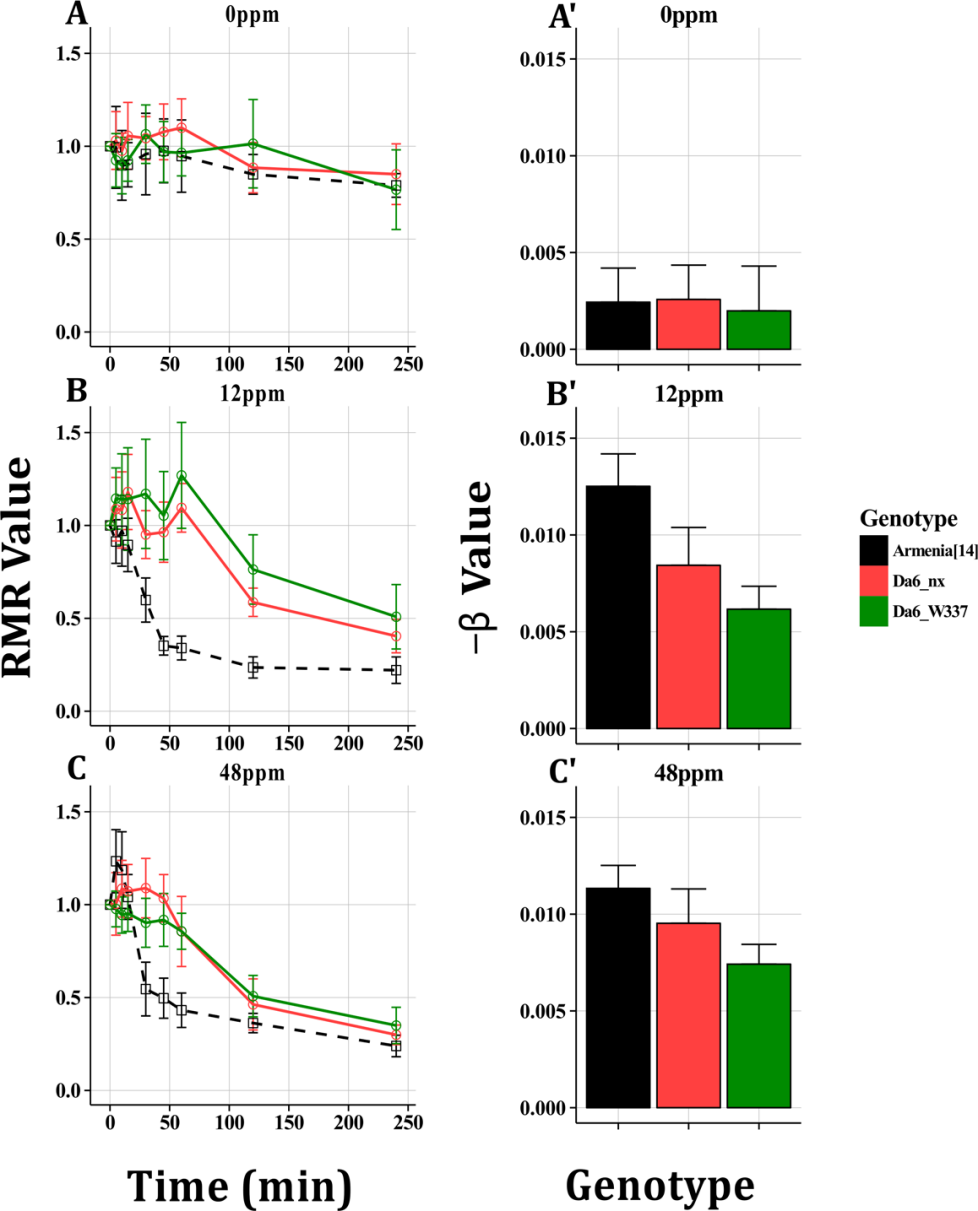
Spinosad Resistance in nAChR Mutants. Armenia^14^, *Dα6* ^W337*^ and *Dα6* ^*nx*^ were tested using the Wiggle Index at 0ppm (A, A’), no significant differences were observed between genotypes. At 12 ppm (B, B’) both *Dα6* mutants displayed significantly delayed response times and responded less in the GLM analysis. However, while *Dα6* ^337*^ differed from Armenia^14^ in End Point RMR value, *Dα6* ^*nx*^ did not. At 48ppm (C, C’), while Response Times were still delayed in Dα6 mutants, End Point RMR values were not significantly different and GLM analysis indicated that only *Dα6* ^337*^ responded more than Armenia^14^. All plots display mean RMR or β values with 95% confidence intervals.

*Dα6* mutant genotypes, *Dα6* ^*nx*^ and *Dα6* ^W337*^, were screened at 2 concentrations of spinosad (12, 48ppm). At each dose both *Dα6* mutants showed delayed Response Times compared to Armenia^14^. At 12ppm *Dα6* ^W337*^ had a larger End Point RMR value when compared to Armenia^14^, but the *Dα6* ^*nx*^ End Point RMR value was not statistically different. At 48ppm there were no differences in End Point RMR values among any of genotypes (Fig 6B and 6C). GLM analysis indicated small but significant differences between Armenia^14^and each *Dα6* mutant at 12ppm, but at 48ppm only *Dα6* ^W337*^ was significantly different from the control (Fig 6B’ and 6C’). Each genotype displayed near identical response profiles at 0ppm, indicating that there were no motility differences in the absence of insecticide (Fig 6A and 6A’).

### Set Three and Four: *Cyp6g1* Overexpression

**Fig 7:**
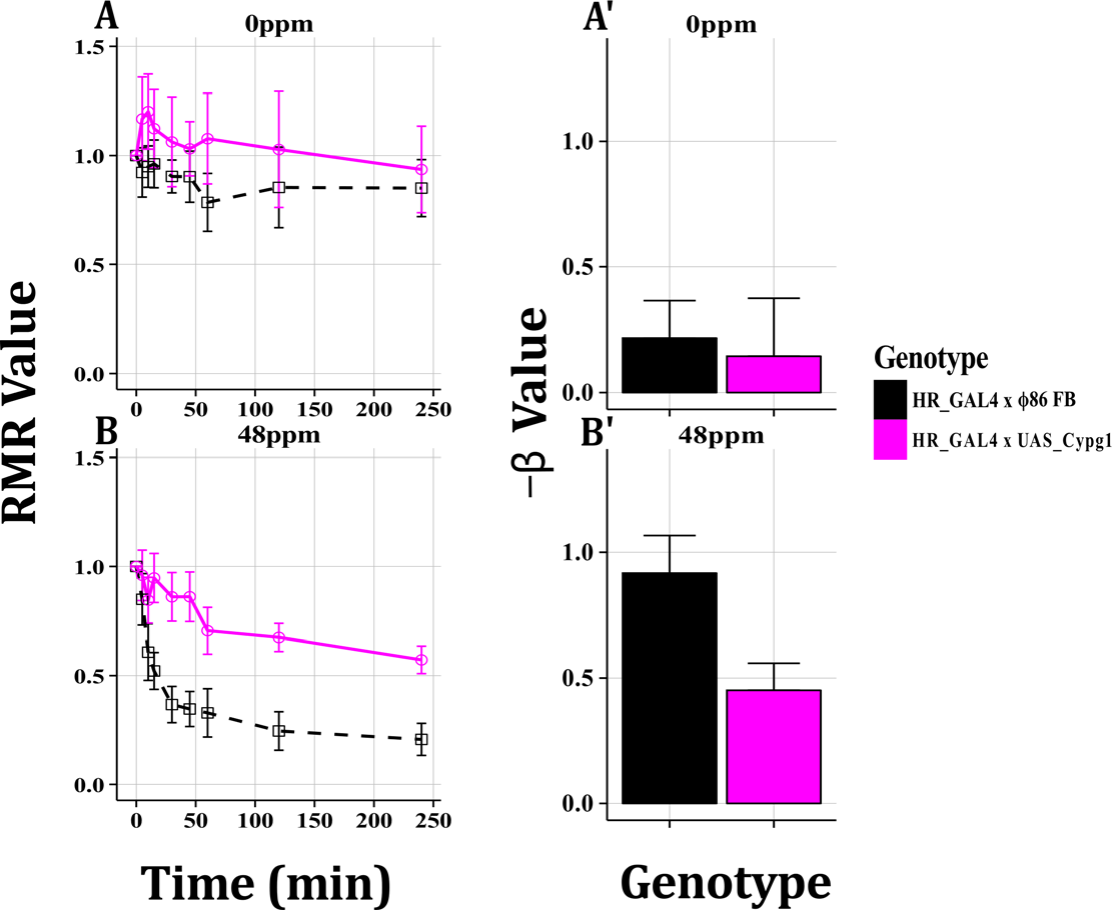
*Cyp6g1* Expression in the Digestive Tissues. *Cyp6g1* overexpression was achieved in the midgut, Malpighian tubule and the fat body using the GAL4-UAS system. At 0ppm (A, A’), no significant differences were observed between genotypes. At 48ppm imidacloprid the overexpression line responded less as measured by Response Time, End Point RMR values and GLM analysis. All plots display mean RMR or β values with 95% confidence intervals.

*Cyp6g1* was overexpressed in the midgut, fat body and Malpighian tubules and tested at 48ppm imidacloprid. Lines that overexpressed *Cyp6g1*, showed a slightly delayed Response Time to imidacloprid than the matched control and showed a significantly higher End Point RMR value, which was supported by GLM analysis (Fig 7B and 7B’). No significant difference was observed at 0ppm (Fig 7A and 7A’).

**Fig 8:**
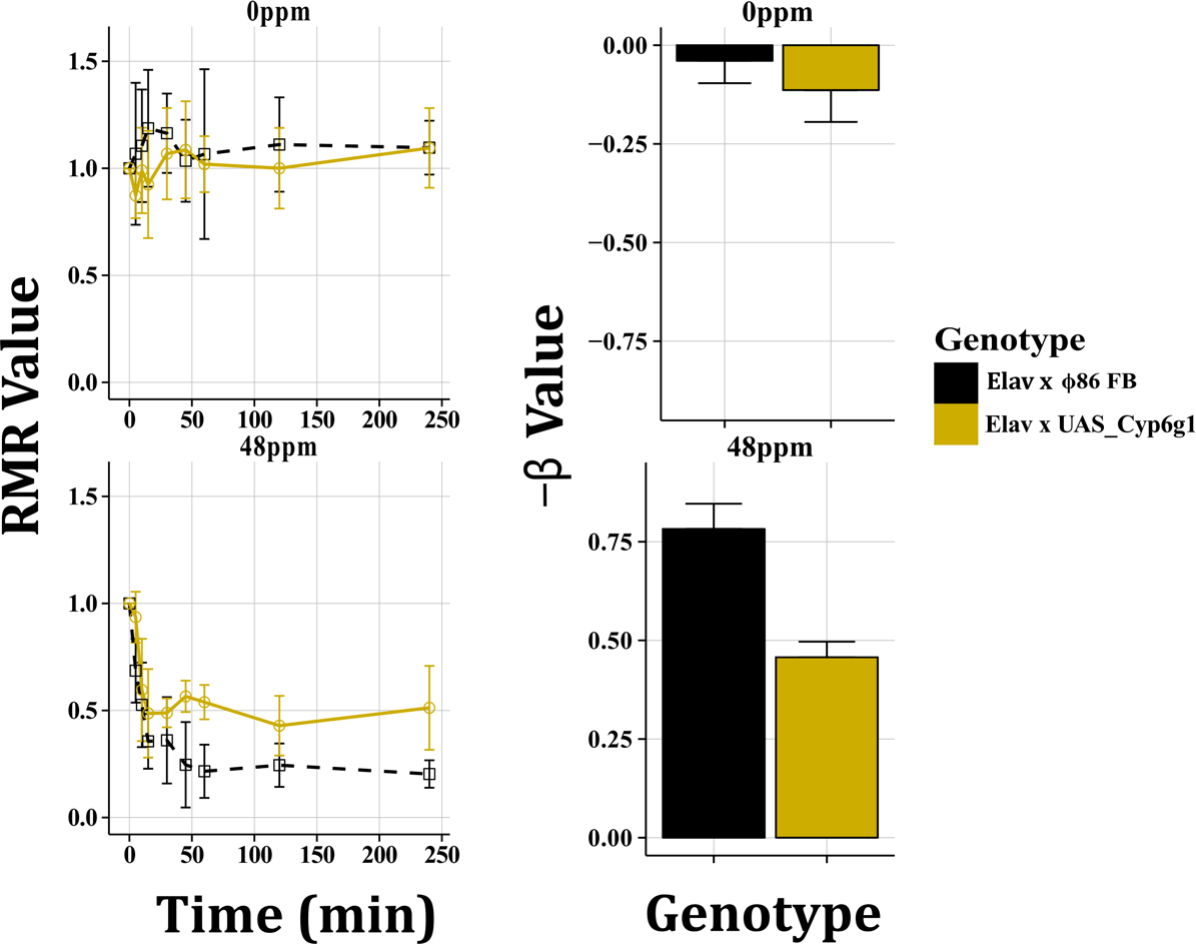
*Cyp6g1* Expression in the Central Nervous System. *Cyp6g1* overexpression was achieved in the CNS using the GAL4-UAS system. At 0ppm (A, A’), no significant differences were observed between genotypes. At 48ppm imidacloprid the overexpression line responded less as measured by Response Time, End Point RMR values and GLM analysis. All plots display mean RMR or β values with 95% confidence intervals.

*Cyp6g1* overexpression in the CNS (Elav x UAS_Cyp6g1 c.f. Elav x Φ86FB) was tested at 48ppm imidacloprid. Elav x UAS_Cyp6g1 displayed a 5 minute delay in Response Time and had an End Point RMR value significantly higher than the control (Fig 8B). GLM analysis also found differences between overexpression and control genotypes (Fig 8B’). Overexpression of *Cyp6g1* in the CNS had less of an impact on all phenotypes than overexpression in the digestive tissues.

## Discussion

### Armenia^14^ Dose Response Profiles

Insect pests impose significant costs on agriculture and human health, but lack of descriptive phenotypes have hindered a deeper understanding of how insects respond and interact with insecticides. Here, a novel method of assessing sub lethal insecticide response in the model insect *D*. *melanogaster* is described, in part, by testing a wild type strain with insecticides of different MoA classes. This strain (Armenia^14^) responded in two distinct ways depending on which of the 4 insecticides was used. Although each of these insecticides belongs to a distinct MoA class, characteristics common between classes may partially explain the types of response profiles observed. Imidacloprid and spinosad target distinct nicotinic acetylcholine receptor subtypes found in the CNS, ivermectin targets ligand gated chloride channels in the same tissue [42], and chlorantraniliprole binds to the ryanodine receptor in muscle cells [43]. Because, imidacloprid, ivermectin, and spinosad all target receptors in the CNS, they must therefore cross the blood-brain barrier. The molecular weight of xenobiotics has been shown to influence their rates of diffusion [44] and transport [45] into the CNS. This could explain the differences in Response Time observed in the WI. Spinosad and ivermectin have molecular weights of 732 Da and 875 Da respectively, while imidacloprid is relatively smaller with a weight of 255 Da. Chlorantraniliprole, though of intermediate size (430 Da), would not be affected by this phenomenon due to its targeting of the more easily accessible ryanodine receptor in muscle cells [43].

### Target Site Mutants

For each set of target site mutants used in this study, significant differences were detected in insecticide response for resistant mutants compared to their matched controls using the WI. Following the addition of imidacloprid, there was always a reduction in RMR values. *Dα1* and *Dβ2* mutants displayed significantly higher End Point RMR values and lower β values than did Armenia^14^ (Fig 5). In contrast, *Dα6* mutants tested with spinosad displayed similar End Point RMR values to Armenia^14^, but their Response Time was significantly delayed (Fig 6). These same mutants were previously found to be more resistant in mortality assays, and it was hypothesized that this was due to a decreased ability of the insecticide to bind its primary target [7,12]. While both mortality and motility are complex phenotypes, these data do link specific alleles to insecticide response phenotypes. Resistance levels observed in mortality assays, however, do not correlate perfectly with the data presented here. For example, *Dα1* ^*M4*^ showed less response to imidacloprid than *Dβ2* ^*L351Q*^ as measured by all three WI phenotypes even though the latter has higher resistance in mortality assays [46]. The partial overlap in the mortality and motility phenotypes for these mutants suggest that different aspects of insect/insecticide interactions are being detected by each assay. These differences could be partially explained by differences in the concentrations of insecticides, stages of development and exposure methods in the two assays. The mortality assays discussed here, were performed on first instar larvae reared to adulthood on solid media dosed with insecticide with much of the mortality occurring at the first instar stage. In contrast, the WI used third instar larvae, exposed for a short time (240 minutes) in liquid media.

The *Dα6* ^*nx*^ mutant, which has no detectable *Dα6* expression, still responds to spinosad in the WI and has similar End Point RMR values when compared to Armenia^14^ and *Dα6* ^W337*^ even though the two *Dα6* mutants have far higher mortality LC_50_ value (Fig 5; Fig 8). To date *Dα6* orthologues are the only genes for which a role in spinosad target site resistance has been demonstrated [17,47]. The *Dα6* ^*nx*^ data presented here suggest that spinosad may also bind to at least one additional target impacting motility. Early mode of action studies indicated that other ligand gated chloride channels may also be spinosad targets [48]. No mutations were found in the *Dα6* orthologue in a spinosad resistant strain of *Musca domestica* [49], but there is evidence that spinosad can bind to *M*. *domestica* α6 [7]. The WI has allowed us to make this significant observation, not detected using classical mortality studies.

There is the potential that baseline differences in motility in the absence of insecticide may confound any motility responses measured by the WI. In the current study the only genotypes to show such differences were the imidacloprid target site mutants (*Dα1* ^*M4*^ *Dβ2* ^*L351Q*^ *)* with each mutant moving significantly less than Armenia^14^ at time point 0 (S1 Fig). Despite lower initial WI values, in the longer term the WI values of the mutants were higher than Armenia^14^. Therefore, even in the absence of any correction for motility differences in the insecticide free control, the WI was still able to differentiate target site mutants. However, only matched genetic backgrounds were tested in the current study. It is therefore possible that comparing field populations may restrict the WI to describing alleles of large effect as is the case with mortality testing [24]. However, quantitative genetics approaches including genome wide association studies may help overcome this obstacle (See section 4.4).

### Cyp6g1

The impact of imidacloprid on motility was reduced significantly when *Cyp6g1* was overexpressed in the digestive tissues (Fig 6). These data correlate with those previously reported for mortality assays [10]. As imidacloprid targets nAChRs in the CNS, the response of larvae may be an indicator of the levels of imidacloprid and/or toxic metabolites in the CNS at a given time. Hence, the data presented here could be explained if the overexpression of *Cyp6g1* reduces the effective concentration of imidacloprid in the CNS. This hypothesis is supported by data from metabolic studies conducted under the same exposure conditions employed here (3^rd^ instar larvae exposed to imidacloprid in 5% sucrose solution). Hoi et al. [50] used Twin Ion Mass Spectrometry to show that increased *in vivo* expression of *Cyp6g1* in the digestive tissues leads to a large increase in the metabolism of imidacloprid to toxic metabolites that are rapidly excreted. As a consequence, when *Cyp6g1* is overexpressed in the metabolic tissues, significantly lower concentrations of imidacloprid and metabolites are retained in the body with access to the CNS.

The impact of imidacloprid on motility was also significantly reduced when *Cyp6g1* was overexpressed in the CNS (Fig 7). While the capacity of *Cyp6g1* to provide neuroprotection has not been previously reported, a role has been proposed for a Cytochrome P450, *Cyp6cm1*, in *Tribolium castaneum* [9].

One advantage of using the WI in combination with the GAL4-UAS system is that it provides the capacity to determine the route(s) that an insecticide follows to arrive at its target. It is theoretically possible that under the conditions used for the WI, that the insecticides tested may have entered the body orally, reaching the CNS via the digestive system, or through the cuticle, gaining direct access to the CNS via the hemolymph. Cuticular thickness has been cited as a resistance mechanism in adult *D*. *melanogaster* [51]. The large difference in RMR values at early time points (5 minutes; Fig 7B) when *Cyp6g1* is overexpressed in digestive tissues indicates that a significant portion of imidacloprid reaches the CNS via the digestive system, probably due to oral ingestion. The path that insecticides and their metabolites follow in larvae could be further studied by using the WI in combination with Twin Ion Mass Spectrometry [50] and the GAL4-UAS system to examine the role of different families of genes such as those encoding transporter or cuticular proteins.

### The Utility of the WI

While only the response of *D*. *melanogaster* was tested in this study, this method offers several insights into insecticide biology in pest species. *D*. *melanogaster* has been frequently used to elaborate on resistance mechanisms in matched genetic backgrounds. For example, the contribution of individual pest genes to resistance has been measured by driving the expression of genes encoding metabolic enzymes and targets in *D*. *melanogaster* [6,9,52]. Beyond the transgenic expression of pest genes in *D*. *melanogaster*, there is also the possibility that the methods used in this study may be modified for use in some other insect species. A similar exposure method has already been used to study drug responses in the parasitic nematode *Haemonchus contortus* (38). While the exposure method described here (24 well plates, chemical concentrations etc.) may be *D*. *melanogaster* specific, the ImageJ algorithm itself only measures the average change in light intensity of pixels in a video. Thus, in theory a variety of different exposure conditions may be used so long as the organism under investigation can be differentiated from its background.

Several technical aspects of the WI make it an attractive option for assessing an insecticide response. Analysing motility is a relatively cheap, high-throughput process when compared with other methods. The WI setup requires only an inexpensive video camera, a source of even light and free downloadable software (See Materials and Methods). Typical mortality assays are measured in adults by analysing how many adults survive insecticide exposure after a given interval or in larvae by measuring what proportion of individuals develop to adulthood [19,53]. The WI measures insecticide response earlier in the life cycle of the organism and significant differences can be observed between genotypes in as little as 5 minutes. The ease of sampling (filming) allows for a nearly unlimited number of time points that can be analysed in a single run. While in this study three parameters were used to quantify insecticide response, many other features of the response profile could be analysed. These factors make the WI suitable for screening large numbers of genotypes.

One of the distinct advantages of using *D*. *melanogaster* is the ability to implicate individual genes in a phenotype by using previously generated genotypes from stock centres such as Vienna *Drosophila* RNAi Centre [54], the Bloomington Deficiency Kit [55] and the MiMic library [56]. Each stock in these collections contains a defined genetic change in a common background, much like the genotypes discussed in the current study. However, other *Drosophila* specific resources use genetic variation similar to those observed in field settings to implicate alleles in complex traits. The *Drosophila* Genetic Reference Panel is a collection of fully sequenced homozygous stocks derived from one field population [57,58]. By phenotyping the collection and performing a subsequent genome wide association study, alleles of potentially very small effect can be associated with a complex trait, such as sub-lethal insecticide response, as has been done for other traits [59,60]. A similar resource, the *Drosophila* Synthetic Population Resource, relies on a synthetic populations derived from several fully sequenced founder individuals [61] and has previously been used to describe the genetic basis for nicotine resistance [62]. The use of these resources, in combination with the WI may lead to the identification of previously undescribed genes influencing insecticide ingestion, metabolism, transport, excretion and those encoding targets.

## Conclusion

The WI quantifies insecticide response by measuring motility and is a valuable addition to the current array of bioassays. By measuring the motility of larvae as they are being exposed, a more complete understanding of this response can emerge. Using insecticides of distinct classes and validation with previously characterized metabolic and target site resistant *D*. *melanogaster*, we have demonstrated that this assay is both sensitive and rapid, while providing novel biological information.

## Supporting Information

S1 Fig WI Value Plot. The uncorrected responses (WI values) for imidacloprid resistant alleles during exposure to 48ppm imidacloprid over A) 240 minutes and B) 30 minutes. Despite lower starting values, the WI Value prior to correction are still clearly capable of discriminating between the known resistant alleles and susceptible strain (Armenia^14^).
(TIF)

S1 Script The Wiggle Index Script. Written in imagej macro language, this script will iterate over a folder of image sequences and will return a sub-folder full of heat maps and a table of WI values. These images and values correspond to the motility of larvae in one well in each image sequence in the folder.
(TXT)

S1 Table Media Recipes. Recipes for both maize meal media (A) and grape juice plates (B) used in this study.
(XLSX)
