# Supplementary figures and images for: The Wiggle Index: An Open Source Bioassay to Assess Sub-Lethal Insecticide Response in Drosophila melanogaster
Source: PLoS One. 2015 Dec 18;10(12):e0145051. doi: 10.1371/journal.pone.0145051 (PMC4684293; doi:10.1371/journal.pone.0145051)

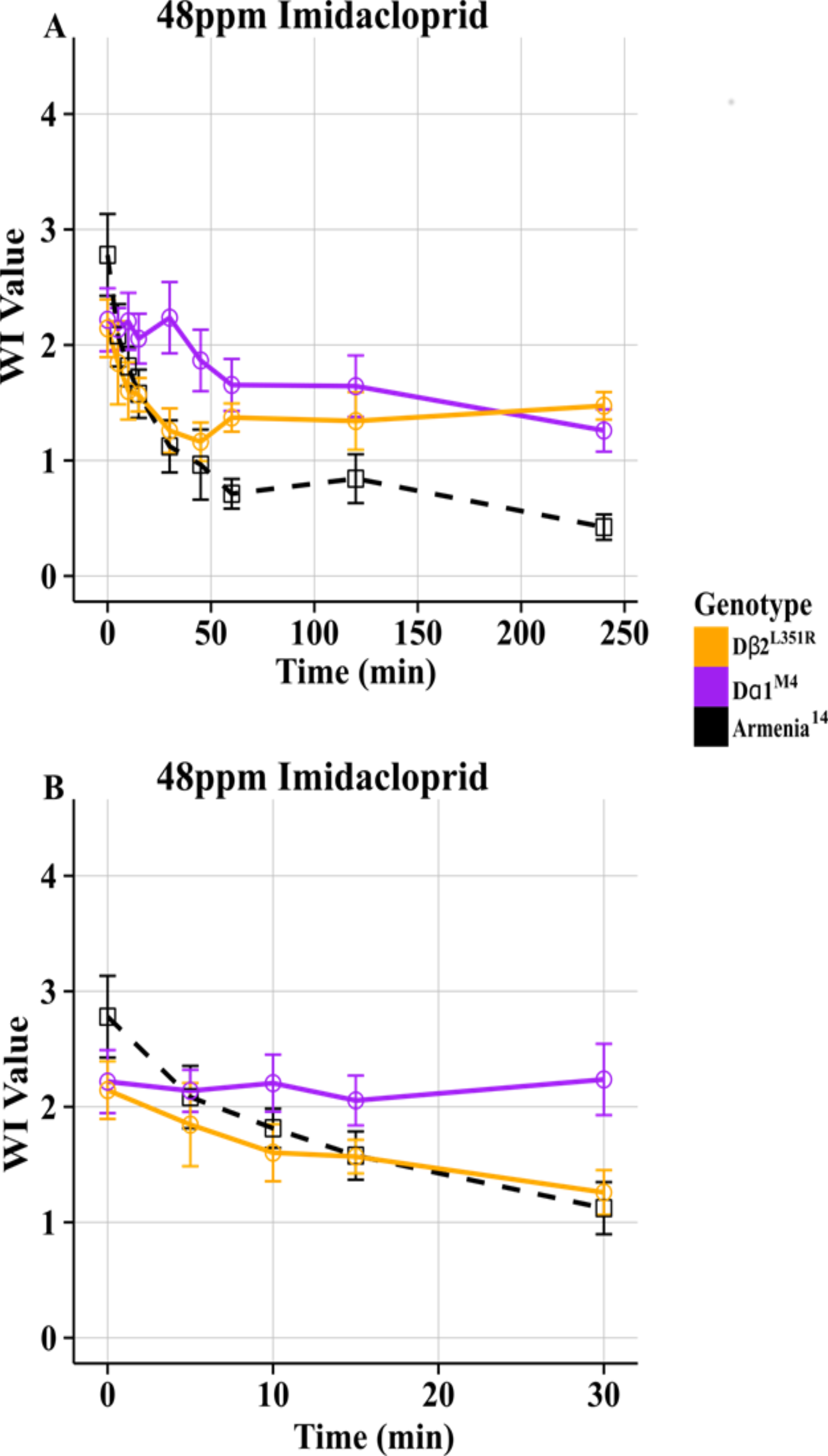

Supplement: S1 Fig — The uncorrected responses (WI values) for imidacloprid resistant alleles during exposure to 48ppm imidacloprid over A) 240 minutes and B) 30 minutes. Despite lower starting values, the WI Value prior to correction are still clearly capable of discriminating between the known resistant alleles and susceptible strain (Armenia14). (TIF) [file pone.0145051.s001.tif]
